# Novel Biodegradable 3D-Printed Analgesics-Eluting-Nanofibers Incorporated Nuss Bars for Therapy of Pectus Excavatum

**DOI:** 10.3390/ijms23042265

**Published:** 2022-02-18

**Authors:** Kuo-Sheng Liu, Wei-Hsun Chen, Chen-Hung Lee, Yong-Fong Su, Yen-Wei Liu, Shih-Jung Liu

**Affiliations:** 1 Department of Thoracic and Cardiovascular Surgery, Chang Gung Memorial Hospital at Linkou, Taoyuan 33305, Taiwan; liuks@me.com (K.-S.L.); m7631@cgmh.org.tw (W.-H.C.); 2 Division of Cardiology, Department of Internal Medicine, Chang Gung Memorial Hospital at Linkou, Chang Gung University College of Medicine, Taoyuan 33305, Taiwan; chl5265@gmail.com; 3 Department of Mechanical Engineering, Chang Gung University, Taoyuan 33302, Taiwan; nickeja2005687@gmail.com; 4 Bone and Joint Research Center, Department of Orthopedic Surgery, Chang Gung Memorial Hospital at Linkou, Taoyuan 33305, Taiwan; anglevvings@gmail.com

**Keywords:** pectus excavatum, 3D printing, biodegradable Nuss bar, polylactide, analgesics

## Abstract

A novel hybrid biodegradable Nuss bar model was developed to surgically correct the pectus excavatum and reduce the associated pain during treatment. The scheme consisted of a three-dimensional (3D) printed biodegradable polylactide (PLA) Nuss bar as the surgical implant and electrospun polylactide–polyglycolide (PLGA) nanofibers loaded with lidocaine and ketorolac as the analgesic agents. The degradation rate and mechanical properties of the PLA Nuss bars were characterized after submersion in a buffered mixture for different time periods. In addition, the in vivo biocompatibility of the integrated PLA Nuss bars/analgesic-loaded PLGA nanofibers was assessed using a rabbit chest wall model. The outcomes of this work suggest that integration of PLA Nuss bar and PLGA/analgesic nanofibers could successfully enhance the results of pectus excavatum treatment in the animal model. The histological analysis also demonstrated good biocompatibility of the PLA Nuss bars with animal tissues. Eventually, the 3D printed biodegradable Nuss bars may have a potential role in pectus excavatum treatment in humans.

## 1. Introduction

Pectus excavatum (PE), also termed “funnel chest”, is a posterior depression of the sternum and adjacent costal cartilages and is responsible for more than 90% of congenital chest wall malformations. The source of PE may originate from unbalanced excessive growth in the costochondral regions. PE patients can manifest chest wall discomfort, exercise intolerance, and tachycardia. In severe cases, PE may also lead to cardiopulmonary impairment and physiological limitations. Previous studies have demonstrated that patients with asymmetric PE possess shorter ribs on the more severely depressed side of the defect. More than 43% of patients with PE possess a family history of the condition. PE is considered a multifactorial inheritance; however, the exact genes implicated in this process remain unclear [1].

Up to the present, the minimal incursive therapy of PE, also named the Nuss process, has been widely used for patients who need surgical correction of PE [2,3]. By means of two tiny incisions on the lateral sides of the chest, an introducer is advanced through the posterior area to the sternum and ribs in addition to through the anterior area to the heart and lungs. A curved stainless-steel bar is slid beneath the sternum along the incisions at the chest side. The introduction of the pectus bar is guided by a thoracoscope, which is inserted via another small skin incision. Once an appropriate position of the pectus bar is achieved, the bar is turned 180 degrees such that the convexity of the bar faces the sternum thus lifting the sternum upwards. Finally, a fixation mechanism is applied to the end of the bar to prevent bar displacement [4]. Tall and/or old patients or patients demanding substantial correction may acquire two or more bars, while additional incisions (more than two incisions) may also be created during surgery. Although the Nuss process is considered as “minimally incursive”, postoperative pain management remains challenging, which in turn demands multi-mode pain control involving epidural anesthetics, narcotics, and non-steroidal anti-inflammatory drugs [5]. In addition, due to its non-degradable characteristic, the metallic Nuss bar requires a second operation for removal at 2–4 years post-implantation.

An ideal Nuss bar for the repair of PE should possess several characteristics: (1) possess good mechanical strengths to correct the chest wall, (2) deliver adequate and sustainable analgesics to the target site for pain relief, (3) be biodegradable after serving its purpose so that a secondary surgery to remove the bar is not needed, and (4) be biocompatible with human tissue so that the material breakdown process would not result in any tissue irritation.

Polylactide (PLA), a biodegradable polymer with good strength, has been widely researched as an excellent biomaterial for load-bearing implants such as fracture fixation devices [6,7]. The material has also demonstrated its instrumental importance as a three-dimensional (3D) printable biopolymer. Meanwhile, poly(lactic-co-glycolic acid) or PLGA presents sustainable drug-eluting capacities that provide controlled delivery of various pharmaceuticals and biomolecules (such as growth factors) for enhancing bone healing, extended discharge of antimicrobial agents for infection control, and biomimetic mats for tendon/ligament repairs [8].

In this work, we designed and exploited novel analgesics-loaded biodegradable Nuss bars using 3D printing and electrospinning techniques. We assumed that successful PE treatment can be effectively achieved using a biodegradable polylactide (PLA) Nuss bar.

## 2. Results

### 2.1. In Vitro Characterizations

The degradation behavior of PLA Nuss bars was investigated. After submersion in buffered mixtures for various times, no evident dimensional variations were noted for the PLA Nuss bars. The mean maximum bending strengths of the bars decreased somewhat over time, namely 375.7 N, 361.9 N, 348.1 N, 341.2 N, 336.3 N, 337.6 N, 332.4 N corresponding to months 0 to 6 (N = 3). Meanwhile, the bending strengths of the retrieved bars from the animals at different times were also measured. The results suggest that the maximum bending strengths (306.5 N at one month, 287.2 N at two months, and 280.3 N at three months) after implantation of PLA bars was reduced in the animals as the time course of the experiment progressed.

**Figure 1:**
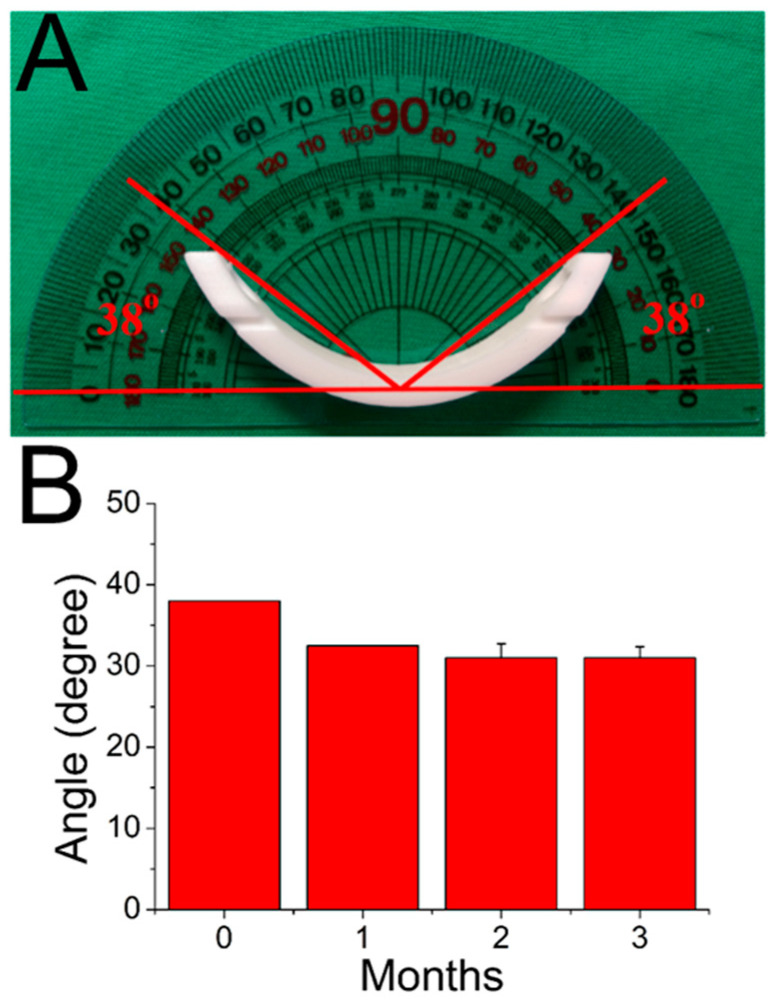
Deformations of PLA bars with time after being implanted in the animals. (**A**) The measured angle of deformation, which is 38° before implantation, (**B**) angles of deformation with times. (*p* > 0.05). Data are presented as mean ± standard deviation.

The in vitro and in vivo molecular weight variations of PLA materials in the 3D printed bars were also characterized using a gel permeation chromatograph. Table 1 shows the variation in in vitro molecular weight distribution over time. While there was no obvious degradation that occurred in the first four months, the molecular weights of the PLA presented considerable reductions after submersion in buffered mixtures after five months (*p* < 0.05). Table 2 displays the molecular weight variation of PLA bars in vivo. The bars had begun to degrade significantly (*p* < 0.05) one month after implantation in the rabbits. Obviously, the bars degraded more rapidly in vivo than in vitro. Furthermore, as illustrated in Figure 1, the PLA bars became somewhat deformed after being implanted in the animals, mainly due to the creeping behavior of polymeric materials when subjected to the constraining force from the chest chamber. The creep behavior leveled off at three months.

### 2.2. In Vivo Animal Studies

**Figure 2:**
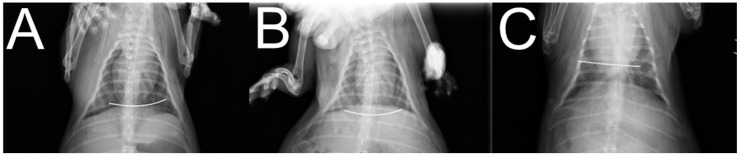
X-ray images of implanted PLA Nuss bars, (**A**) 1 month, (**B**) 2 months, (**C**) 3 months post-implantation (a metal wire was inserted at the core of the bars for easy identification).

Figure 2 illustrates the X-ray images of the implanted PLA Nuss bars at post-implantation one, two, and three months. The radiographic examination results suggest that implanted PLA remained in the original positions without obvious migration.

**Figure 3:**
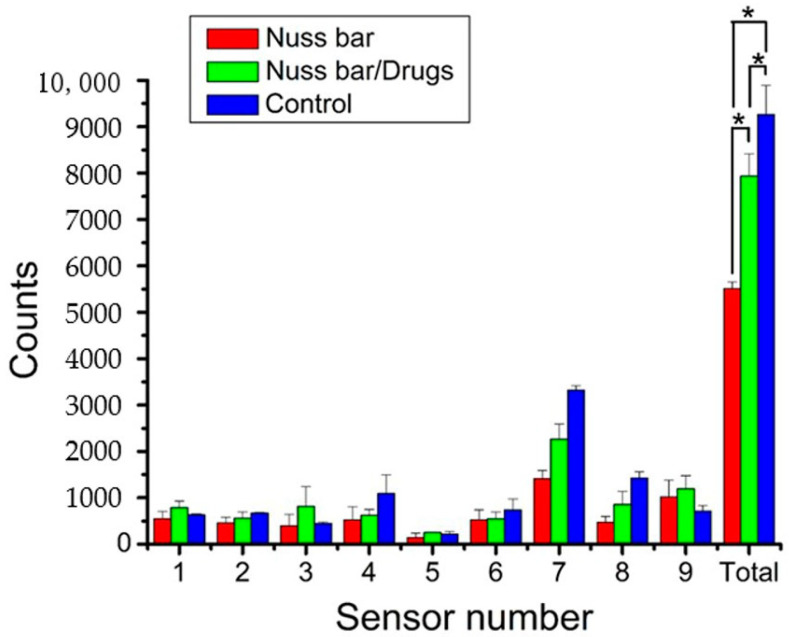
Sensor counts of the animals in the activity cage (* *p* < 0.05). Data are presented as mean ± standard deviation.

Figure 3 displays the trigger counts of the three groups over the six-day post-surgical period. The total counts thus acquired were 5512 ± 148, 7935 ± 478, and 9265 ± 628 for PLA bar group, PLA bar/drugs group, and control, respectively. Additionally, the animals in all groups exhibited the greatest trigger counts at sensor 1, corresponding to the location of food and water supplies. The number of triggers at all other sensors was higher than that of sensor 5, indicating persistent migration along the cage wall. In addition, the rabbits in both Group A (PLA bar only) and Group B (PLA/analgesics bar) showed inferior post-implantation activities compared to that of Group C (*p* < 0.05) mainly due to the pain associated with the trauma of surgery. Meanwhile, the animals in Group B exhibited a greater number of sensor triggers than Group A (*p* < 0.05), demonstrating the efficacy of analgesics loaded on PLA for pain relief.

**Figure 4:**
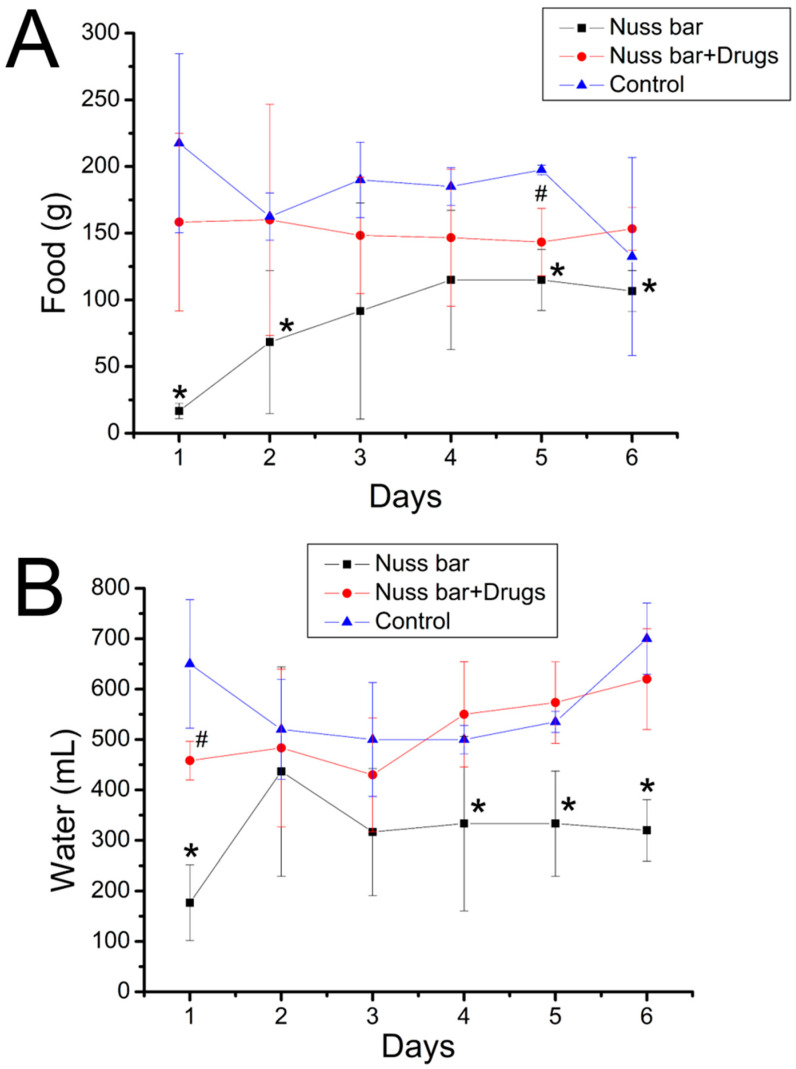
(**A**) Food and (**B**) water intake of the animals in various groups (# *p* < 0.05, Nuss bar/drugs versus control; * *p* < 0.05, Nuss bar versus control). The animals implanted with a PLA bar had significantly less food and water intake; however, the animals received implantation of analgesic-loaded PLA bars that exhibited food and water intake comparable to that of the control. Data are presented as mean ± standard deviation.

**Figure 5:**
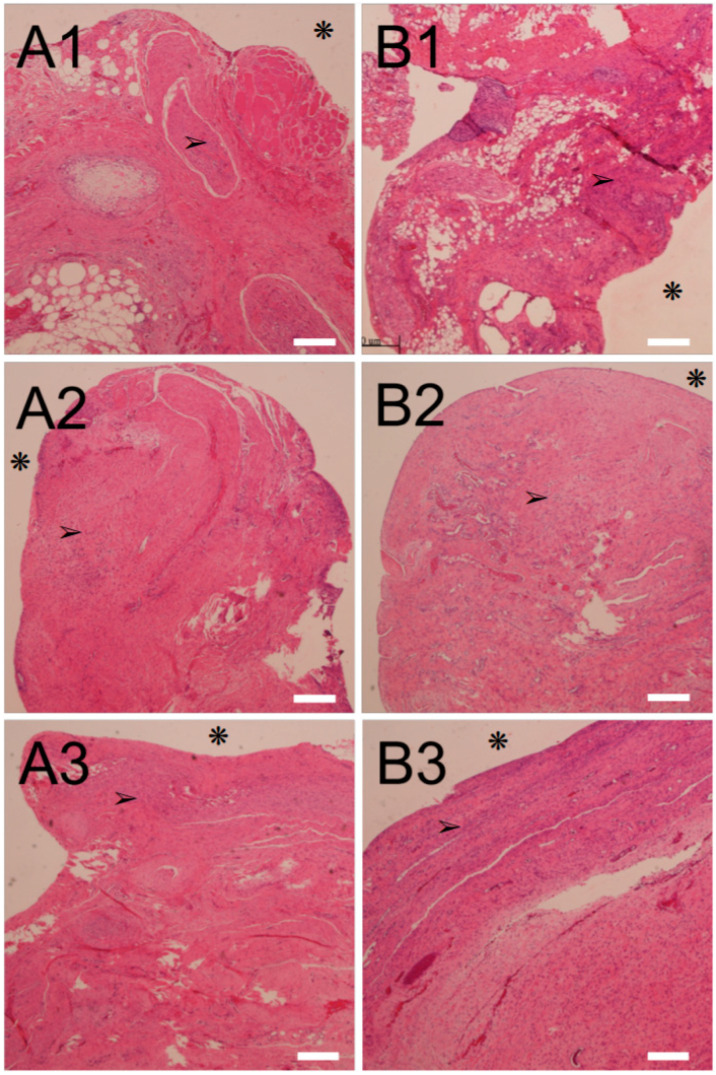
Histological results with hematoxylin and eosin (H&E) stain. (**A1**–**A3**) are the images of PLA Nuss bars at 1, 2, and 3 months post-implantation, respectively, while (**B1**–**B3**) are those of PLA Nuss combined with analgesic-loaded PLGA nanofibers. There was no residual prosthetic material in these pictures. Asterisks (❋) indicate the site of bar position; arrows (➢) indicate inflammatory leukocytes. (Scale bar = 300 μm).

Figure 4A,B shows the food and water intake of the animals. The animals implanted with a PLA bar had significantly less food and water intake (*p* < 0.05); however, the animals received implantation of analgesic-loaded PLA bars that exhibited food and water intake comparable to that of the control. This finding further demonstrates that the lidocaine- and ketorolac-incorporated nanofibers could effectively ease the pain associated with the implantation of the PLA Nuss bars. Finally, the histological examination of the peri-Nuss bar tissue of Groups A and B (Figure 5) showed comparable mild inflammatory leukocyte infiltration throughout the study period, indicating mild inflammatory response of the experimental animals to the implanted biodegradable PLA bars, with or without the analgesic-loaded PLGA nanofibers.

## 3. Discussion

In this work, we developed biodegradable Nuss bars with PLA materials as the backbone and PLGA as the vehicle for analgesic delivery to the target site, using 3D printing and electrospinning technologies. 3D printing is the creation of a 3D article from a computer-aided design model [9,10]. 3D printing can be used to establish medical devices/implants that are made-to-order for a patient’s designated anatomy or a particular surgical procedure, rendering the process potentially more efficacious than if performed with a mass-produced device. Customized, operative- and/or patient-designated 3D-printed tools and implants are highly feasible. While conventional devices/implants may require several weeks to design and manufacture, 3D printing provides the possibility of minimizing the time needed to fabricate implants. Furthermore, 3D printing is often cheaper, and sometimes faster, than standard fabrication methods, indicating that manufacturers can rapidly design, manufacture, and verify medical device prototypes. Integrated with the possibility of personalized medical devices, the 3D printing approach can provide on-demand individual implants, such as the pectus bars used in the Nuss procedure.

PLA is a biodegradable, hydrolyzable aliphatic semi-crystalline polyester produced through the direct condensation reaction of lactic acid monomers. PLA materials range from amorphous to semi-crystalline polymers, possessing a glass transition temperature of 60 to 65 °C, a melting temperature of 130 to 180 °C, and a tensile modulus of 2.7 to 16 GPa [7]. PLA degrades inside the body within six months to two years and has been widely used as medical implants in the form of plates, screws, pins, and rods [11,12,13]. The gel permeation chromatography (GPC) analysis of the retrieved PLA bars, as shown in Table 2 suggests that the bars underwent degradation over time. This slow degradation process is advantageous for a support device since the process gradually shifts the load to the body during the recovery process. Additionally, the creep phenomenon was observed for the PLA Nuss bars post-implantation (Figure 1). To increase the bending strengths and minimize any possible bar creeping, the PLA of high molecular weights or composites [14,15] reinforced with fillers such as carbon [16] or graphene [17] may be used. Among distinct biomaterials, PLGA has demonstrated itself great potential as a drug delivery carrier and as scaffolds for tissue engineering [8,18]. The degradation rate of PLGA is related to the LA:GA monomers’ ratio, and the copolymer with 50:50 ratio exhibits the faster degradation (approximately 1–2 months) [19,20,21]. Our previous study incorporated analgesic-eluting nanofibers onto metallic Nuss bar and achieved the sustained and effective release of lidocaine and ketorolac for post-surgery pain relief for over 10 days (28.3 μg/mL and 17.8 μg/mL, respectively, for lidocaine and ketorolac at day 10) [22]. In addition, we used ethanol for the disinfection of PLA bars. Despite no obvious influence was noted on the bars, the disinfection could still affect the drug release behavior. For future applications, the PLA bars/PLGA nanofibers may also be sterilized by ethylene oxide or gamma irradiation [23] before implantation. The effects of these sterilizations on the printed bars and spun nanofibers should be further investigated.

Almost all patients who undergo surgery experience acute post-surgical pain. Nevertheless, <50% of the patients receive appropriate post-surgical pain relief [24]. Post-surgical pain, when not well controlled, may result in unfavorable physiological reactions and increase the hazards of post-surgical complications, in addition to allowing post-surgical pain to continue. Pain also causes an increase in the cost of medical care and hinders wound healing and successive restoration to normal life. Post-surgery pain management is thus a primary concern for both surgeons and patients [25]. In this work, the analgesic-eluting nanofibers were integrated with the PLA Nuss bars. Our previous study [22] investigated the influence of metal bars on animals’ activity, and found that the trigger count for metal bar implanted rabbits was 6184 ± 323. The bare PLA bar implanted animals exhibited inferior trigger counts (5512 ± 148) than the metal bar deployed animals (*p* < 0.05), possibly due to the greater thickness of PLA bars (PLA bar: 4.14 mm, metal bar: 3.0 mm) that induced more severe pain in the rabbit’s chest cavity. The outcome of activity analysis (Figure 3) and food/water intake (Figure 4) of the rabbits demonstrated the effectiveness of the loaded lidocaine/ketorolac in relieving post-surgical pain. Furthermore, the histological results showed no obvious adverse responses by the tissues. This procedure provides advantages in terms of pain management after PLA bar implantation. 3D-printed biodegradable Nuss bars may have a potential role for the treatment of pectus excavatum in humans.

One major limitation of the present study is that a non-PE animal model was used. Whether the PLA bars will perform differently in a funnel chest is not known. Further evaluation of the PLA bars in a PE model is necessary to answer this question. Additionally, the long-term outcome of implanted PLA bars, such as the potential fracture of the bars due to polymer degradation, is unclear. The characterization of remained analgesics on the fibers as well as activity of these analgesics should be conducted. Finally, the relevance of our findings to humans with PE remains unrevealed and needs to be further explored. These will be the topics of our future studies.

## 4. Materials and Methods

### 4.1. 3D Printing of Degradable Nuss Bars

The degradable Nuss bars were prepared using a fused deposition modeling (FDM) printing method for commercial PLA filaments (weight average molecular weight (Mw) of 110,000 g/mol, Prolink Microsystems Corp., Taipei, Taiwan) on a 3D printer. The filament has a diameter of 1.75 ± 0.2 mm. In the printing process, the printer extruded melted polymeric material from a nozzle (possessing an internal diameter of 200 μm) at an extrusion speed of 30 mm/s. The nozzle head, which was set at a temperature of 230 °C, heated the material and adjusted the flow. Once the material was pushed out of the nozzle, it hardened and deposited layers. Two microprocessor-controlled step-motors were used to shift the printing head and regulate the flow of molten polymers.

**Figure 6:**
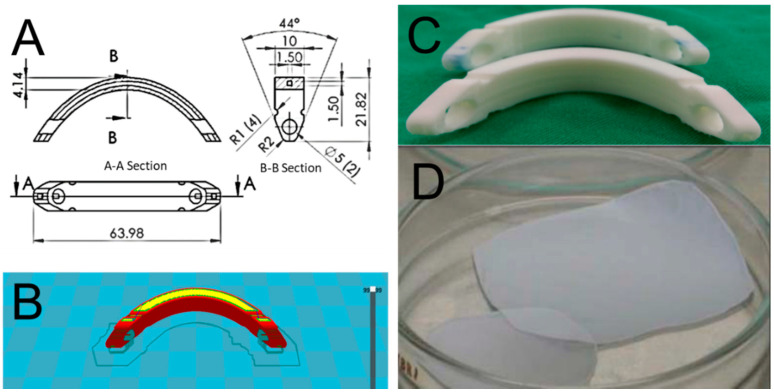
(**A**) Layout and dimensions of curved Nuss bars, (**B**) computer-aided design of the bars, (**C**) Three-dimensional (3D)-printed polylactic (PLA) Nuss bars, (**D**) analgesics-loaded poly(lactic acid-co-glycolic acid) or PLGA nanofibers. (unit: mm).

Figure 6A shows the layout and dimension of the Nuss bars. A tiny tunnel was designed along the whole length of the bar to allow insertion of a metal wire, allowing easy identification of the bars under X-ray examination. Before printing, the Solidworks (Waltham, MA, USA) and the Cura (Ultimaker B.V., Geldermalsen, The Netherlands) software were employed to create the code (Figure 6B) that monitors the entire printing procedure. Figure 6C shows a photo of the printed PLA bars. The average time for printing a bar was approximately 2 h.

### 4.2. Assessment of Fabricated PLA Bars

The in vitro variation of molecular weights in the PLA bars was assessed via submerging the bars in a phosphate buffered mixture at 37 °C. Specimens were retrieved from the mixture at 1–6 months (in increment of one month). After dehydration for 24 h, the molecular weights of PLA bars were evaluated using a Waters gel permeation chromatograph (Milford, MA, USA).

The mechanical strengths of the 3D printed PLA Nuss bars were also characterized after being submerged in the phosphate buffer solution for various time points. Three-point bending tests of the bars were completed on a tensile test machine (Lloyd-Ametek, Largo, FL, USA). During the measurements, the bars were compressed by the top loading pin at an increasing rate of 5 N until the end of the test. The ultimate strength and elongation at break were monitored. The evaluation was done in triplicate (N = 3) for each bar.

### 4.3. Lidocaine and Ketorolac-Eluting Nanofibers

Analgesics-eluting nanofibers, which were previously developed and fabricated in our lab by electrospinning of commercially available PLGA, were employed. Lidocaine and ketorolac were employed as the analgesics. Figure 6D shows a photo of the electro-spun nanofibrous membranes, which had a thickness of approximately 0.12 mm and discharged sustained lidocaine and ketorolac for over 10 days, as shown in our previous study [22].

### 4.4. Surgical Procedure, Animal Care and Assessments

Eighteen New Zealand white rabbits (weight ranging from 2.2 to 2.5 kg) were used in this study. The entire animal-related process acquired approval from Chang Gung University (CGU105-052), and all enrolled rabbits were cared for according to the regulations of the Department of Health and Welfare, Taiwan.

**Figure 7:**
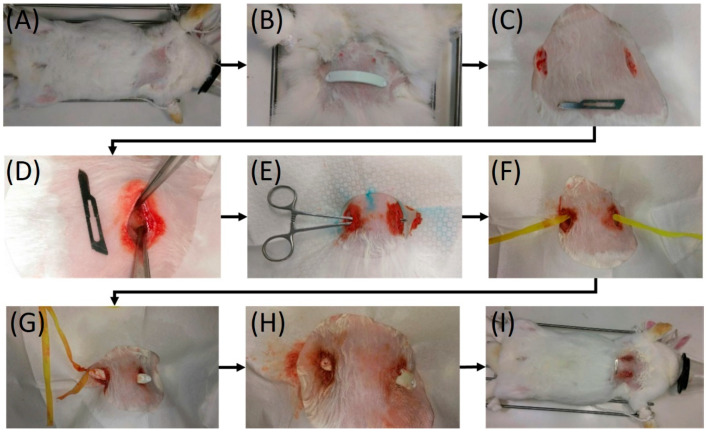
Surgical procedure for the implantation of biodegradable Nuss bars. Notes: (**A**) Ventral side of the experimented rabbit. (**B**) Biodegradable Nuss bar before implantation. (**C**) Two 1.5 cm skin incisions were made in the bilateral chest. A No. 15 blade was used as reference. (**D**) The wound was deepened to the subpectoral muscle plane. (**E**) A substernal tunnel was created with a Pean clamp. (**F**) A polyester tape was introduced into the substernal tunnel. (**G**) The polyester tape was tied to the end hole of the biodegradable Nuss bar, which was then guided into the substernal tunnel. (**H**) The polyester tape was removed, and the Nuss bar was then rotated 180 degrees to accommodate the chest wall anatomy. (**I**) Completion of the surgical procedure.

The rabbits were separated into three distinct groups: (1) PLA bar group (Group A, PLA only, N = 6), (2) PLA bar with analgesic-eluting nanofibrous membrane group (Group B, PLA/PLGA-lidocaine-ketorolac group, N = 6), and (3) control (Group C, received no implantation, N = 6). Before implantation, the bars were disinfected with ethanol at a concentration of 75%. All 18 rabbits underwent general anesthesia via isoflurane respiration. Figure 7 shows the outline of the entire surgical procedure. The anesthetized rabbits (Groups A and B) were placed in a supine position. The ventral chest wall was dehaired, disinfected, and covered in an aseptic way. Two incisions in the skin, 1.5-cm each, were created in the bilateral chest near the anterior axillary line at the level of approximately 2 cm on top of the xiphoid process. The incision was teased apart. A Pean clamp was employed to pierce through the intercostal muscle, impelling into the substernal plane, and penetrating out of the intercostal muscle at the contralateral side. A polyester tape was grasped using the Pean clamp. By retrieving the Pean clamp, the tape was taken into the substernal tunnel and fastened to the end hole of the PLA bar. The bar was directed into the substernal tunnel with the convexity confronting the dorsal side by applying a mild pulling force on the plastic tape. As soon as it was in position with an equivalent length of the bar protruding on each side, the bar was revolved by 180 degrees such that the convexity faced the sternum. The end holes of the PLA bar were fastened to the surrounding tissues via resorbable sutures. The incisions were then occluded.

**Figure 8:**
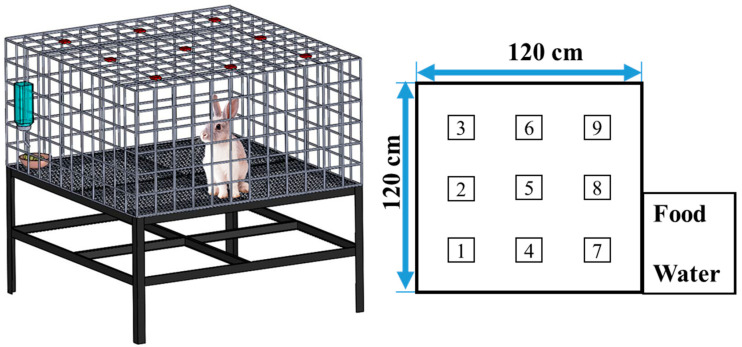
Layout and dimensions of the animal activity cage.

After completing the surgical procedure, the animals received post-operatory analgesics (Ketorolac, intramuscular, 1 mg/kg per 12 h) in the first 24 h. Thereafter, the general activity of each animal was assessed using an activity cage with dimensions of 120 cm (W) × 120 cm (L) × 60 cm (H). As illustrated in Figure 8, nine diffusion-scan type photoelectric switch sensors (HP100-A1, Azbil Corp., Tokyo, Japan) located on top of the cage were used to record the migrations of the animal in the cage. When the animal migrated from one region to the other, the sensor at the “approaching” region was then triggered. The microcomputer was used to monitor the total trigger number for each rabbit for six consecutive days.

Furthermore, the daily food/water intakes of the animals were recorded. The 12 rabbits that received surgery underwent radiologic examination at 1-, 2-, and 3-months post-implantation.

### 4.5. Biomechanical and Histological Assays

All 12 rabbits were euthanized by intravenous injections of lethal dosage of lidocaine at postoperative 1, 2, and 3 months for PLA bar retrieval for mechanical strength measurements and a histological examination.

The mechanical properties of the retrieved PLA bars were characterized by the three-point bending test described earlier in Section 4.2. At 1, 2, and 3 months post-implantation, tissues surrounding the bars were sampled. Hematoxylin and eosin staining were used for histological evaluation. Sectioned slides were assessed by an independent pathology doctor, blinded to the control and experiment groups. Five images were evaluated for each group.

### 4.6. Data Analysis

We used the paired *t*-test for statistical evaluation between different two-group combinations with SPSS software (Version 12.0; SPSS Inc., Chicago, IL, USA), with statistical significance set at *p* < 0.05. All data are presented as mean ± standard deviation.

## 5. Conclusions

We successfully developed a novel hybrid biodegradable Nuss bar model for the surgical correction of PE and a reduction of the associated pain during treatment. The experimental results show that the integration of PLA Nuss bar and PLGA/analgesics nanofibers can successfully enhance the therapy in a PE rabbit model. The histological analysis also demonstrated good biocompatibility of the PLA bars with animal tissues. The 3D-printed biodegradable Nuss bars may have a potential role in the treatment of PE in humans.

## Figures and Tables

**Table 1:** In vitro molecular weight variations of polylactic acid (PLA) Nuss bars. (*, *p* < 0.05).

Month	M_n_ (g/mol)	M_w_ (g/mol)	M_z_ (g/mol)	M_z+1_ (g/mol)
0	82,324	131,285	209,010	332,034
1	79,299	130,040	208,501	323,281
2	73,919	125,981	215,028	404,728
3	75,556	126,283	203,429	317,216
4	76,162	124,987	202,498	321,703
5	63,335	103,871 *	159,125	235,957
6	49,520	82,292 *	124,499	185,040

M_n_: number average molecular weight, M_w_: weight average molecular weight, M_z_: z average molecular weight, M_z+1_: z + 1 average molecular weight. (The values are the mean).

**Table 2:** In vivo molecular weight variations of PLA Nuss bars. (*, *p* < 0.05).

Month	M_n_ (g/mol)	M_w_ (g/mol)	M_z_ (g/mol)	M_z+1_ (g/mol)
0	82,324	131,285	209,010	332,034
1	77,761	119,825 *	185,333	281,082
2	67,566	103,177 *	156,349	233,279
3	55,364	90,472 *	138,254	206,018

M_n_: number average molecular weight, M_w_: weight average molecular weight, M_z_: z average molecular weight, M_z+1_: z + 1 average molecular weight. (The values are the mean).
